# Mismatch Repair System Gene Expression in FFPE Samples from Breast Cancer Patients

**DOI:** 10.3390/medsci13040256

**Published:** 2025-10-31

**Authors:** Ricardo Quidute, Patricia Quidute, Matheus Moreira Perez, Carlos Henrique F. Peiró, Glaucia Luciano da Veiga, Beatriz da Costa Aguiar Alves, Fernando Luiz Affonso Fonseca

**Affiliations:** 1 Laboratório de Análises Clínicas do Centro Universitário FMABC, Santo André 09060-650, SP, Brazil; ricardoquidute@gmail.com (R.Q.); patriciaslquidute@gmail.com (P.Q.); matheusmpontoperez@gmail.com (M.M.P.); caiquefoncesca@gmail.com (C.H.F.P.); glaucia.veiga@fmabc.br (G.L.d.V.); beatriz.reis@fmabc.br (B.d.C.A.A.); 2 Departamento de Ciências Farmacêuticas, Universidade Federal de São Paulo (UNIFESP), Diadema 09972-270, SP, Brazil

**Keywords:** breast cancer, neoplasia, FFPE, repair system, *hMSH2*, *hMSH6*, gene expression

## Abstract

**Background/Objectives**: Cancer characteristics are mainly due to mutations in genes that regulate key pathways. One mechanism for identifying and correcting these mutations is the Mismatch Repair System. Given the lack of data on MMR in breast cancer FFPE samples, this study aims to examine *hMSH2* and *hMSH6* gene expression in FFPE tumor biopsies. **Methods**: One hundred patients with breast cancer were included in this study, from which tumor samples were obtained. Expression of the *hMSH2* and *hMSH6* genes was evaluated by qPCR. **Results**: Out of 100 tumor samples, 89 were suitable for molecular analysis. Of these, 10 (11.23%) expressed the *hMSH2* gene and 4 (4.5%) expressed the *hMSH6* gene. No associations were found between *hMSH2* and *hMSH6* expression and tumor staging, HER2, ER, PR, or Ki-67 expression. **Conclusions:** Our results suggest that the expression of the proposed markers is decreased in breast tumorigenesis.

## 1. Introduction

Breast cancer (BC) is the leading cause of cancer-related mortality among women in both developed and developing countries. According to the World Health Organization, in 2020, there were 2.3 million new diagnosed cases and 685,000 breast cancer-related deaths [1]. Breast cancer (BC) is marked by abnormal growth and proliferation of breast tissue cells, which can invade nearby tissues and metastasize organs like the brain, bones, liver, and lungs [2]. The risks associated with BC vary based on individual and disease characteristics, such as age, family history, hormonal status, lifestyle, mutations in tumor-suppressor genes, and tumor type, size, and location [3]. Identification of the cancer biomarkers Estrogen Receptor (ER), Progesterone Receptor (PR) and the oncoprotein Human Epidermal Growth Factor Receptor 2 (HER2) aid in characterizing tumor type, predicting prognosis and defining treatment, since they are important therapeutic targets of chemotherapeutic drugs [4].

Ki-67, a proliferating cell nuclear antigen, is another important indicator for prognosis and therapeutic selection, measurable in formalin-fixed, paraffin-embedded (FFPE) tumors stored for many years. An increased percentage of malignant cells positive for this marker is associated with decreased survival rates in breast cancer patients [5]. In addition to Ki-67, numerous other molecular and genetic markers are used to predict disease development, prognosis, and therapeutic response. Research continues to focus on discovering new markers to improve the chances of curing breast cancer and enhancing patients’ quality of life.

The characteristics common to different types of cancer, such as evasion of apoptosis and growth inhibitory factors and the ability to invade and metastasize, are mainly due to the accumulation of pathological genetic variations (mutation) in genes responsible for regulating these pathways [6]. One of the mechanisms responsible for identifying and correcting these variations is the Mismatch Repair System (MMR) [7].

The MMR is composed of a group of proteins that recognize and repair pairing errors, deletions and insertions resulting from failures in DNA polymerase activity during DNA replication or caused by genomic instability related to malignant transformation of cells [2]. Some of the main proteins of this family are “human MutL Homolog 1” (hMLH1), “human MutS Homolog 2” (hMSH2), “human MutS Homolog 3” (hMSH3), “human MutS Homolog 6” (hMSH6) and “PMS1 Homolog 2” (PMS2). These homologs interact with each other, forming heterodimers with different functions [8]. The MutSα complex, a heterodimer formed by the proteins hMSH2 and hMSH6, acts in the correction of errors involving only one nitrogenous base. Errors involving two to six nitrogenous bases are corrected by the MutSβ complex, a heterodimer formed by the hMSH2 and hMSH3 proteins [9].

The genetic instability characteristic of many cancers is primarily due to the loss of function of the MMR [10]. This dysfunction, caused by epigenetic events and germline mutations in the *hMLH1* and *hMSH2* genes (components of the MutLa and MutSα complexes), leads to increased errors in DNA pairing and genetic variations. This loss of function is linked to diseases and comorbidities, such as recurrent and poor-prognosis oral squamous cell carcinoma [11] and Lynch Syndrome (LS) [7]. LS raises the risk of various cancers, particularly hereditary nonpolyposis colorectal cancer, due to a mutagenic phenotype called microsatellite instability (MSI), which results in the accumulation of frameshift mutations in tumor suppressor genes [12].

Given the importance of the MMR in cancer development, several studies have investigated defects in its function or the differential expression of its proteins. The MutSα complex, composed of MSH2 and MSH6, plays a central role in mismatch recognition within the MMR. While the full MMR pathway includes other key components—such as MLH1 and PMS2 (which form the MutLα complex)—our study focused specifically on the MutSα heterodimer due to its direct involvement in the initial recognition of base–base mismatches and small insertion/deletion loops [8,9]. And due to the lack of data on MutSα complex expression in breast cancer tissue, this study aims to examine *hMSH2* and *hMSH6* gene expression in formalin-fixed, paraffin-embedded tumor biopsies from breast cancer patients.

## 2. Materials and Methods

### 2.1. Patients

This is a cross-sectional exploratory study that included breast cancer patients from the Instituto de Mama do Cariri (Juazeiro do Norte—Ceará, Brazil). Informed consent was obtained from each participant. Inclusion criteria were women over 30 with breast cancer confirmed by anatomopathological examination. Women with non-neoplastic breast comorbidities or other cancers or chronic diseases like HIV, hepatitis, and diabetes were excluded. The study was approved by the Centro Universitário FMABC Research Ethics Committee (protocol no 346712/19, approval in August 2019) and conducted from September 2019 to July 2020. Informed consent was obtained from all subjects involved in the study. Breast tumor biopsies obtained from the patients were fixed in 10% buffered formalin, embedded in paraffin and cut using a micrometer into sections (10 μm) for histopathological, immunohistochemical and molecular analyses. Patients with earlier-stage disease received adjuvant chemotherapy, while those with more advanced-stage disease received neoadjuvant chemotherapy. All tumor samples were obtained before the start of treatment, so chemotherapy did not interfere with the gene expression results.

### 2.2. Isolation of Total RNA from Paraffinized Tumors

RNA was isolated from a total of 5 sections of 10 μm slices from paraffinized tumors previously analyzed by a pathologist from each patient using the RNeasy^®^ FFPE Kit (Qiagen, cat. no. 73504, Hilden, Germany), according to the manufacturer’s recommendations. Only samples containing at least 70% tumor cells were selected for RNA extraction and gene expression analysis. RNA concentration was measured by spectrophotometry (NanoVue Plus—GE Health Care, Buckinghamshire, UK). Integrity of isolated RNA and expression normalization values were evaluated by qPCR amplification of the reference gene *GAPDH* [13].

### 2.3. Reverse Transcription and qPCR

cDNA synthesis was performed using 100 ng of total RNA with the QuantiNova Reverse Transcription Kit (Qiagen, cat. no. 205413, Hilden, Germany), according to the manufacturer’s recommendations. Expression of the *hMSH2* and *hMSH6* genes was evaluated by qPCR. Specific primers for *hMSH2* (F- CCTTGTAAAACCTTCATTTGATCC and R- ATCCAAACTGTGCACTGGAA, 157 bp), *hMSH6* (F- GTGAAACTGCCAGCATACTCA and R- GCCGCTCCATCAAATGTTGC, 94 bp) and *GAPDH* (F- GACCACAGTCCATGCCATGA and R- CAGCTCAGGGATGACCTTGC, 148 bp) genes were designed using Primer3 Input 0.4.0 software, available at https://primer3.ut.ee/ (accessed on 24 October 2024). Amplifications were carried out in duplicates using an Applied Biosystems 7500 Real-Time PCR System (Applied Biosystems, Foster City, CA, USA) with the Quantitec SYBR Green PCR kit (Qiagen, Cat No. 204143, Hilden, Germany) and 0.2 μM of each specific primer in a final volume of 15 μL. The thermal conditions included an initial hot start phase at 95 °C for 10 min, followed by 45 cycles of 95 °C for 15 s and 60 °C for 25 s.

Detection of target gene expression for each patient was evaluated using the 2^−ΔCt^ formula [14] and was associated with the clinical and anatomopathological characteristics of the patients.

### 2.4. Statistical Analysis

For qualitative variables, absolute and relative values were used. For quantitative variables (Shapiro-Wilk, *p* < 0.05), the mean, minimum, maximum, median, and 25th and 75th percentiles were calculated. Chi-square tests, Student’s *t*-test, and the Mann-Whitney test were used to analyze associations between clinical and anatomopathological variables and the characteristics of patients expressing *hMSH2* and *hMSH6*. The chi-square test was applied to analyze the association between *hMSH2* and *hMSH6* expression. A 95% confidence level was used for all analyses, with Stata version 11.0 (StataCorp LLC, College Station, TX, USA) as the software.

## 3. Results

### 3.1. Patients’ Characteristics

One hundred patients with breast cancer were included in this study and were grouped and classified according to age, tumor stage, presence of estrogen, progesterone and HER2 receptors, chemotherapy treatment and Ki-67 expression (Table 1).

### 3.2. Expression of Target Genes

Out of 100 tumor samples, 89 were suitable for molecular analysis. Among these, 10 (11.23%) expressed the *hMSH2* gene, and 4 (4.5%) expressed the *hMSH6* gene. The presence or absence of *hMSH2* and *hMSH6* gene expression in the 89 samples was linked to clinicopathological parameters, as detailed in Table 2. No associations were found between the presence or absence of *hMSH2* and *hMSH6* expression and tumor staging, presence of HER2, ER and PR and Ki-67 expression, but an association was found between the presence or absence of *hMSH2* expression and chemotherapy (*p* = 0.002). Among the 86 chemotherapy patients, 8 (9.30%) expressed *hMSH2*, while 78 (90.70%) did not. Of the 3 patients who did not undergo chemotherapy, 2 (66.66%) expressed *hMSH2*, and 1 (33.34%) did not. No association was found between *hMSH6* expression and chemotherapy.

An association was made between the expression of *hMSH2* and *hMSH6* in the 89 patients included in the analysis, as shown in Table 3.

As can be seen, of the total, 10 patients showed expression of hMSH2 and 4 showed expression of hMSH6. Only one patient exhibited simultaneous expression of both genes, suggesting potential activation of the MutSα DNA repair complex. The vast majority of cases (76 patients) showed no expression of either gene, as indicated by the complete absence of Ct values and amplification curves. In 12 cases, expression was detected for at least one gene (9 patients expressed only hMSH2, 3 patients expressed only hMSH6 and 1 patient expressed both genes). The chi-square test resulted in a *p*-value of 0.796, indicating no statistically significant association between the expression of hMSH2 and hMSH6 in this cohort. This suggests that the expression of one gene does not predict or correlate with the expression of the other, reinforcing the possibility that other DNA repair pathways may be independently activated.

## 4. Discussion

Given the central role of the MutSα complex (MSH2/MSH6) in the initial recognition of DNA mismatches, this study focused on evaluating the expression of hMSH2 and hMSH6 in breast cancer tissues. In this study, only women over 30 years old were included. Breast cancer in women under 30, although rare, may exhibit distinct biological characteristics. The decision to include only women over 30 years of age was based primarily on practical and epidemiological considerations. In the study region, cases of breast cancer in very young women are infrequent and were not available in sufficient numbers to allow for meaningful analysis. Therefore, a cut-off of 30 years was adopted to focus on the age group most representative of the breast cancer population treated at our institution. Of the 89 usable samples, 10 expressed the *hMSH2* gene and 4 expressed the *hMSH6* gene. The low expression frequency of these genes can be explained by the fact that most cases (84%) were low-stage cancer (stages 0, IA, IIA and IIB). Since these genes are considered tumor suppressors, in many types of cancer their increased expression may be correlated with tumor aggressiveness, metastasis, recurrence and decreased survival [11].

Among the patients, only one showed simultaneous expression of both *hMSH2* and *hMSH6* genes, suggesting activation of the MutSα DNA repair complex and errors in only one base. In contrast, isolated expression of either *hMSH2* or *hMSH6* in other patients indicates the activation of different repair complexes. Most studies on these markers focus on protein expression through immunohistochemical assays, with increased protein expression believed to result from higher cell proliferation rates [15]. Overexpression of certain MMR proteins, caused by genetic and epigenetic alterations that increase gene copies, transcription, and translation rates, can have both beneficial and harmful effects. The most frequently overexpressed genes are *hMSH2* and *hMSH6* [16]. However, decreased expression of repair genes is also observed in various cancers. An immunohistochemical analysis of hMLH1 and hMSH2 proteins in 44 upper urinary tract urothelial carcinoma biopsies showed that *hMSH2* was absent in 25 cases (57%), with 21 (84%) being high-grade, and hMLH1 was absent in 8 cases (18%), with 7 (88%) being high-grade [17]. Another study found the absence of hMSH2, hMSH6, and hMLH1 proteins in upper urinary tract urothelial carcinoma cases with microsatellite instability or mutations in these genes [18]. Similar findings were reported by Uraki et al. [19], who linked decreased protein and gene expression of *hMSH2* and *hMSH6* to higher rates of pituitary adenoma proliferation. While many studies emphasize the role of increased expression of MMR components in cancer progression, our findings highlight the low expression frequency of hMSH2 and hMSH6 in early-stage breast cancer. This may reflect a distinct molecular context, as most of our samples (84%) were from low-stage tumors (0–IIB), where the DNA repair system may not yet be significantly upregulated or destabilized.

This study found no associations between *hMSH2* and *hMSH6* expression and clinicopathological variables such as staging, HER-2, PR, ER, Ki-67, and age. Amaral-Silva et al. [20] in an immunohistochemical analysis of repair proteins in samples of oral tumors, demonstrated an association between simultaneous overexpression of *hMSH2*, *hMSH3* and *hMSH6* with an increased probability of recurrence. Decreased expression of hMSH2, hMSH3 and hMSH6 proteins was also demonstrated when compared to tumor-free tissue, suggesting system regulation in carcinogenic processes and allowing an increase in the occurrence of genetic variations. However, as is the case with our results, no correlation was observed between expression levels and other prognostic variables of the disease, such as the Ki-67 marker. Likewise, Khilko et al. [21] demonstrated that expression of hMLH1 and hMSH2 proteins in 211 cases of BC was not related to any clinical characteristics of the patients, suggesting that these proteins do not play an essential role in most cases of BC.

The data from various studies are inconsistent. A yeast test showed that overexpression of *Msh6* can promote cancer-related gene rearrangements, while joint overexpression of *Msh2* and *Msh6* led to genomic instability, increased mutagenicity, and heightened sensitivity to DNA replication inhibitors and genotoxic agents, promoting tumor progression-like effects [16]. A study using immunohistochemistry to assess hMSH2 expression in tumor samples found increased expression in patients with different stages of BC compared to healthy women, which may be explained by MSI and could serve as a prognostic marker for BC [22]. Another study showed decreased *hMSH2* expression in the peripheral blood of BC patients undergoing chemotherapy, suggesting that the decrease, correlated with increased MSI, is due to alkylating agents in chemotherapy drugs [23]. Kappil et al. [24] found an association between decreased expression of repair genes in the mononuclear blood fraction and breast cancer status in 219 women, with those having a family history of BC showing a higher prevalence due to decreased gene expression. Finally, gene expression in both peripheral blood and tumors is associated with treatment response.

Among the breast cancer patients studied, 11.23% expressed the *hMSH2* gene and 4.49% expressed the *hMSH6* gene. No association was found between gene expression and clinical variables, except for *hMSH2* expression and chemotherapy use. The absence or reduced expression of MMR family proteins has been described in some studies using immunohistochemistry, but there is limited data on the expression of these genes in BC biopsies, especially in paraffin samples. From a clinical perspective, understanding patterns of reduced expression might help identify tumors with a silent or non-functional repair profile, which could inform prognosis or responsiveness to DNA-damaging therapies.

This study has some limitations. Although 100 patients were initially included, only 89 samples were suitable for gene expression analysis, which may have limited the statistical power, especially in subgroup analyses. Whereas sample heterogeneity can limit statistical power in small cohorts, it also reflects the clinical diversity of breast cancer and enhances the generalizability of the findings. In this context, the present study provides an initial overview of hMSH2 and hMSH6 gene expression in a real-world patient population. Future studies with larger samples should include stratified analyses by molecular subtype to further explore potential subtype-specific patterns. The low expression frequency of hMSH2 and hMSH6 also restricted deeper correlations with clinicopathological variables. Furthermore, the absence of a true control group is a limitation. Tissues sent for pathological analysis are inherently abnormal and may contain inflammatory or other changes, making them unsuitable as healthy controls. In addition, our study specifically focused on *hMSH2* and *hMSH6* because together they form the MutSα complex, which is directly responsible for the initial recognition of base–base mismatches and small insertion/deletion loops. While MLH1 and PMS2 (MutLα complex) also play a key role in the downstream repair steps, we chose to concentrate on the MutSα dimer given the lack of data regarding its expression in breast cancer tissues and its central role in mismatch recognition. Future studies should include a broader panel of MMR genes to provide a more comprehensive picture of the repair pathway.

Although qPCR was used in this study, the analyses were not designed to provide quantitative comparisons of gene expression levels between groups. Due to the characteristics of the samples (FFPE material with variable RNA quality and yield) and the exploratory nature of the study, we adopted a qualitative approach, considering the presence or absence of amplification curves and specific melting peaks as indicators of gene expression. Therefore, we reported the detection of *hMSH2* and *hMSH6* expression in a binary manner (expressed vs. not expressed), rather than presenting quantitative fold changes.

Also, we acknowledge the importance of including a control group for conducting robust comparative analyses. However, it is important to clarify that any breast tissue sample submitted for histopathological examination is, by definition, clinically suspicious and therefore cannot be considered representative of truly healthy tissue. Even benign breast lesions often exhibit inflammatory or reactive changes, which may influence the expression of DNA repair genes such as *hMSH2* and *hMSH6*. The presence of such alterations introduces the potential for bias, making these samples unsuitable as negative controls. Moreover, ethical and practical constraints made it unfeasible to obtain normal breast tissue from healthy individuals for research purposes. As a result, the present study was specifically designed to investigate the expression of *hMSH2* and *hMSH6* exclusively in malignant breast tissue samples. It is worth noting that gene expression analyses using formalin-fixed, paraffin-embedded (FFPE) samples without a control group have been previously conducted and published in the scientific literature. For instance, Wagner et al. [11] analyzed the overexpression of MutSα complex proteins in oral squamous cell carcinoma, and Nielsen et al. [25] compared PAM50 intrinsic subtyping with immunohistochemistry and clinical prognostic factors in tamoxifen-treated, estrogen receptor-positive breast cancer. Both studies were carried out without the inclusion of a healthy control group. Therefore, we believe that the methodological approach adopted in our study is scientifically sound and consistent with established practices in the field.

Another limitation of this study is the lack of clinical follow-up data, which prevented analysis of outcomes such as survival, recurrence, and metastasis. Due to the retrospective design and ethical restrictions, access to detailed patient records was not possible. Therefore, the prognostic significance of *hMSH2* and *hMSH6* expression could not be assessed. Future prospective studies with clinical follow-up are needed to validate these findings.

## 5. Conclusions

Our results suggest that the expression of the proposed markers is decreased in breast tumorigenesis; however, further studies on the repair system may highlight these genes as biomarkers of response to treatment for breast cancer.

## Figures and Tables

**Table 1:** Clinical characteristics of the patients included in this study.

Variables	*n*	%
Stage		
0	2	2
IA	16	16
IIA	34	34
IIB	32	32
IIIA	5	5
IIIB	11	11
HER2		
Negative	62	62
+	20	20
++	6	6
+++	12	12
ER		
Negative	18	18
Positive	81	81
Inconclusive	1	1
BPR		
Negative	21	21
Positive	79	79
Chemotherapy		
No	3	3
Yes	97	97
	Mean (sd)	Min–Max
Age (years)	57.21 (13.73)	49–68
	Median	p.25–p.75
Ki-67 (%)	5	0–20

p.25–p.75: 25th and 75th percentiles.

**Table 2:** Association between the expression of the *hMSH2* and *hMSH6* genes and the clinicopathological data of the patients.

Variable	*hMSH2*		*p* *	*hMSH6*		*p* *
	No Expression ^a^ (%)	Expression ^b^ (%)		No Expression ^a^ (%)	Expression ^b^ (%)	
Stage						
0	1 (100.00)	0 (0.00)	0.967	1 (100.00)	0 (0.00)	0.379
IA	14 (87.50)	2 (12.50)		15 (100.00)	0 (0.00)	
IIA	25 (89,29)	3 (10.71)		25 (89.29)	3 (10.71)	
IIB	25 (86.21)	4 (13.79)		29 (100.00)	0 (0.00)	
IIIA	4 (100.00)	0 (0.00)		4 (100.00)	0 (0.00)	
IIIB	10 (90.91)	1 (9.09)		10 (90.91)	1 (9.09)	
HER-2						
Negative	48 (87.27)	7 (12.73)	0.493	53 (96.36)	2 (3.64)	0.153
+	16 (88.89)	2 (11.11)		18 (100.00)	0 (0.00)	
++	3 (75.00)	1 (25.00)		4 (100.00)	0 (0.00)	
+++	12 (100.00)	0 (0.00)		10 (83.33)	2 (16.67)	
ER						
Negative	17 (94.45)	1 (5.55)	0.384	17 (94.45)	1 (5.55)	0.818
Positive	61 (87.14)	9 (12.86)		67 (95.72)	3 (4.28)	
Inconclusive	1 (100.00)	0 (0.00)		1 (100.00)	0 (0.00)	
PR						
Negative	19 (95.00)	1 (5.00)	0.316	18 (90.00)	2 (10.00)	0.177
Positive	60 (86.96)	9 (13.04)		67 (97.10)	2 (2.90)	
Chemotherapy						
No	1 (33.34)	2 (66.66)	0.002	3 (100.00)	0 (0.00)	0.702
Yes	78 (90.70)	8 (9.30)		82 (95.35)	4 (4.65)	
	Mean (sd)		*p* **	Mean (sd)		*p* **
Age	56.87 (13.92)	63.3 (10.19)	0.162	57.43 (13.82)	61.00 (10.29)	0.613
	Median (95% CI)		*p* ***	Median (95% CI)		*p* ***
Ki-67 (%)	1.0 (0.0; 10.0)	7.5 (0.0; 18.3)	0.681	3.5 (0.0; 10.0)	2.5 (0.0; 60.0)	0.907

* Chi-square. ** Student’s *t*-test. *** Mann-Whitney. 95% CI: 95% confidence interval. ^a^ = complete absence of Ct values or amplification curve; ^b^ = clear amplification curve and a melting peak consistent with the expected melting temperature (Tm).

**Table 3:** Association between *hMSH2* and *hMSH6* gene expression.

Gene	*hMSH2*		*p* *
	No Expression	Expression	
*hMSH6*			
No expression **	76	9	0.796
Expression ***	3	1	

* Chi-square. ** = complete absence of Ct values or amplification curve; *** = clear amplification curve and a melting peak consistent with the expected melting temperature (Tm).

## Data Availability

With the exception of disclosing patients’ identity, the raw data will be made available upon legitimate request to the corresponding author. Clinical data is contained within the Supplementary Materials.

## Supplementary Materials

The following supporting information can be downloaded at: https://www.mdpi.com/article/10.3390/medsci13040256/s1, Table S1: clinical data.

## Abbreviations

The following abbreviations are used in this manuscript:

BC	Breast cancer
ER	Estrogen Receptor
PR	Progesterone Receptor
HER2	Human Epidermal Growth Factor Receptor 2
FFPE	Formalin-fixed, paraffin-embedded
MMR	Mismatch Repair System
hMLH1	human MutL Homolog 1
hMSH2	human MutS Homolog 2
hMSH3	human MutS Homolog 3
hMSH6	human MutS Homolog 6
PMS2	PMS1 Homolog 2
MSI	microsatellite instability
